# Recent Progress of Non-Isocyanate Polyurethane Foam and Their Challenges

**DOI:** 10.3390/polym15020254

**Published:** 2023-01-04

**Authors:** Said El Khezraji, Hicham Ben youcef, Larbi Belachemi, Miguel A. Lopez Manchado, Raquel Verdejo, Mohammed Lahcini

**Affiliations:** 1 IMED-Lab, Faculty of Sciences and Techniques, Cadi Ayyad University, Avenue Abdelkrim Elkhattabi, B.P 549, Marrakech 40000, Morocco; 2 Instituto de Ciencia y Tecnologia de Polimeros (ICTP), CSIC, C/Juan de la Cierva, 3, 28006 Madrid, Spain; 3 Mohammed VI Polytechnic University, Lot 660, Hay Moulay Rachid, Ben Guerir 43150, Morocco

**Keywords:** biobased polyurethane, blowing agent, non-isocyanate polyurethane, polymeric foams, polyurethane foams, self-blowing

## Abstract

Polyurethane foams (PUFs) are a significant group of polymeric foam materials. Thanks to their outstanding mechanical, chemical, and physical properties, they are implemented successfully in a wide range of applications. Conventionally, PUFs are obtained in polyaddition reactions between polyols, diisoycyanate, and water to get a CO_2_ foaming agent. The toxicity of isocyanate has attracted considerable attention from both scientists and industry professionals to explore cleaner synthesis routes for polyurethanes excluding the use of isocyanate. The polyaddition of cyclic carbonates (CCs) and polyfunctional amines in the presence of an external blowing agent or by self-blowing appears to be the most promising route to substitute the conventional PUFs process and to produce isocyanate-free polyurethane foams (NIPUFs). Especially for polyhydroxyurethane foams (PHUFs), the use of a blowing agent is essential to regenerate the gas responsible for the creation of the cells that are the basis of the foam. In this review, we report on the use of different blowing agents, such as Poly(methylhydrogensiloxane) (PHMS) and liquid fluorohydrocarbons for the preparation of NIPUFs. Furthermore, the preparation of NIPUFs using the self-blowing technique to produce gas without external blowing agents is assessed. Finally, various biologically derived NIPUFs are presented, including self-blown NIPUFs and NIPUFs with an external blowing agent.

## 1. Introduction

Polymeric foam materials are important from both industrial and economic perspectives. These materials are lightweight and possess high thermal insulation [1,2], high sound insulation [3,4], resistance to impact, and damping properties, which allow them to fulfil the requirements for a wide range of applications. Polymer foams have been successfully implemented in the automotive industry [5], packaging, electronics [6], building construction [7], bedding [8], and medical applications [9]. Foams require the presence of a gas that is commonly introduced via blowing agents or is generated in situ during polymerization.

Among foams, polyurethanes (PUs) are one of the largest families and have widespread applications in diverse areas because they can be formulated to meet specific requirements depending on their nature, whether flexible or rigid [10,11]. PU foams (PUFs) are produced by polyaddition of isocyanates to polyols in the presence of water, which generates CO_2_ gas. However, the presence of isocyanate, which is manufactured using phosgene, makes this classical method toxic [12,13]. Thus, scientists are studying new ways and methods to manufacture PUF without using this raw material. The polymer obtained via this method is then named non-isocyanate polyurethane (NIPU) or polyhydroxyurethane (PHU) [14,15,16]. 

**Figure 1:**
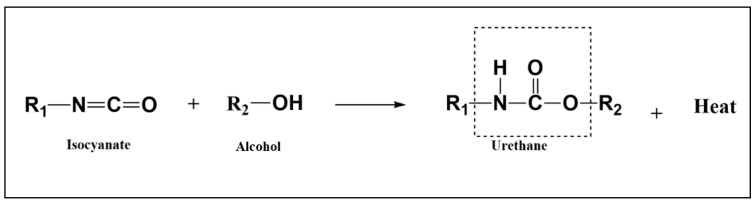
Classical reaction to synthesize urethane linkage adapted from [8].

The basic reaction for the preparation of NIPUF is the aminolysis of cyclic carbonate with a diamine in the presence of a catalyst, with or without an external blowing agent [17]. Figure 1 shows the aminolysis reaction used for the preparation of NIPUF. Polyhydroxyurethane foams have been used with bio-polyols as well as other natural monomers such as glucose [18], vanillyl alcohol [19], and tannin-based compounds [20]. These polymeric foams are prepared using cyclic carbonates (CCs) as well as diamines; foaming occurs via the inclusion of blowing agents or in situ reactions, also known as self-blowing foams.

Recent reviews have reported sustainable routes for the preparation of NIPUs via several methods as well as starting monomers [15,21,22,23]. Here, we report the synthesis procedures and the remaining challenges of NIPUFs using both an external blowing agent or self-blowing reactions.

## 2. General Reaction of Polyurethane and Challenges in Non-Isocyanate Polyurethane

Polyurethanes were originally created by Bayer AG more than 75 years ago, and have a broad variety of molecular structures and polymeric characteristics. Foams (65% of the market), coatings (13%), elastomers (12%), adhesives (7%), and other industries, most notably the biomedical sector (3%), constitute the majority of the PU market [24,25,26]. By 2026, more than $61.5 billion worth of polyurethane foam will be produced worldwide, up to 44% from 2021 [27]. Soft foams, such as those used in pillows or mattresses, to stiff foams, which are mostly found in acoustic insulation materials, require a wide range of properties for commercial applications [7]. PUFs are currently made by exothermically reacting formulations of polyisocyanates and polyols [28]. Isocyanates are often hydrolyzed while CO_2_ is simultaneously produced, resulting in foaming. Thus, when carbon dioxide is released, the polymer dilates, resulting in foams whose properties depend on the composition of the reactive formulation. However, the components of isocyanate and polyisocyanate are both extremely dangerous to human health and cause severe asthma and other respiratory conditions [29,30,31]. Consequently, the U.S. Environmental Protection Agency and EU REACH rules have increased their regulatory pressure on methylene diphenyl diisocyanate and toluene diisocyanate, which are categorized as CMR (carcinogenic, mutagenic, and reprotoxic) substances.

PU foams are produced by two simultaneous processes: polymerization and gelling of polyols with isocyanates and the CO_2_-producing blowing reaction from the hydrolysis of isocyanates. The correct growth of a foam with the necessary features depends on the precise management of the kinetics of these two conflicting processes [32]. The word “PU” refers to the urethane linkage that results from the interaction of the OH (hydroxyl) groups of a polyol and the NCO (isocyanate functional group) groups of an isocyanate to produce polymers. Exothermic in nature, this reaction creates urethane groups, as previously mentioned and shown in Figure 1.

A second reaction takes place between the isocyanate and water to obtain CO_2_, which is the reason for the creation of the cells and, consequently, the formation of the PU foam. This reaction produces carbamic acid, which then breaks down into carbon dioxide. In the polymer precursor, carbon dioxide dissolves and concentrates until it achieves supersaturation, at which time nucleation starts [33].

Despite the yield and ease of preparing PU foams via this method, polyisocyanate toxicity remains a major drawback to overcome. Research effort has been devoted to finding new methods of PUF preparation, excluding the use of isocyanates. Thus, non-isocyanate polyurethanes (NIPU) are appealing and promising materials for the creation of polyurethanes that are environmentally friendly and may be entirely based on renewable raw ingredients. Among the most promising alternatives to the traditional synthesis of PU is the aminolysis reaction between a bicyclic or tricyclic carbonate monomer and a diamine [34]. 

**Figure 2:**
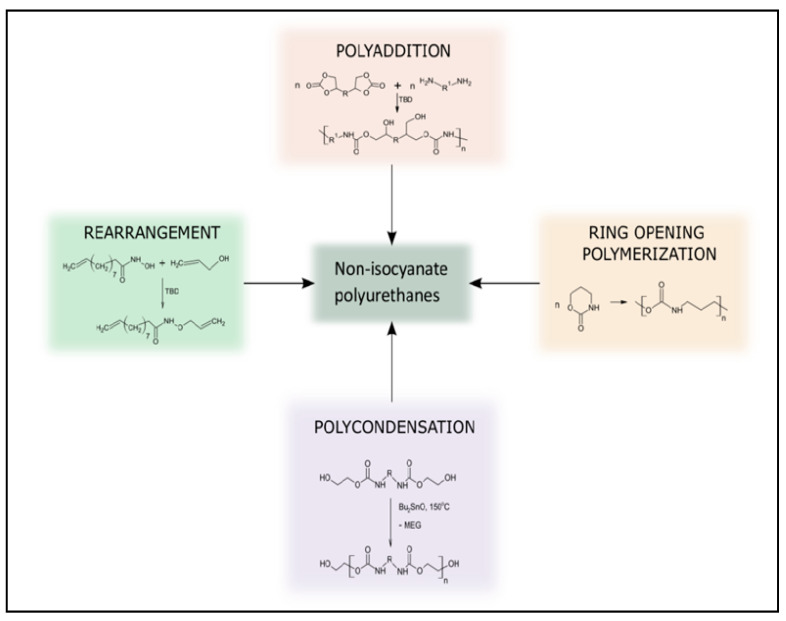
Different pathways used to prepare NIPU. Reprinted from ref [42] with permission from the publisher.

Five-membered cyclic carbonates are desirable monomers because they are assumably non-toxic [35]. The most environmentally friendly method for producing cyclic carbonates from biomass involves the trans-esterification of a bio-based diol with dimethyl carbonate, which is widely regarded as a “green” reagent, or by epoxidizing olefins and reacting the resulting epoxide with carbon dioxide in the presence of a suitable catalyst [36]. After the synthesis of the cyclic carbonate, the aminolysis reaction of the obtained CC with a diamine is the key reaction for the preparation of isocyanate-free polyurethane. Aminolysis or polyaddition of diamines and cyclic carbonate is a direct way to obtain this type of polymer using a suitable catalyst (Figure 2). The formation of every urethane linkage induced the formation of a secondary or primary hydroxyl group. Thus, NIPUs may be alternatively termed polyhydroxyurethanes [37,38]. Other reactions used to obtain NIPU have been explored, but to a lesser extent than polyaddition (e.g., rearrangement, ring-opening polymerization, and polycondensation).

Cyclic carbonates and amine precursors are gaining interest for the synthesis of polyhydroxyurethane. Cyclocarbonates have a significant advantage over several other types of reagents due to their high solubility and boiling temperature [39]. Various types of diamines or polyamines are used in this method, either aliphatic or aromatic amines, which give us a wide choice of NIPUs with different structural, mechanical, thermal, and chemical properties [23,40,41]. Another advantage of this method is the possibility of obtaining bio-based CC monomers by using natural monomers such as vanillyl alcohol or bio-based oils in the CO_2_ fixation reaction.

Polyurethane foams are synthesized by reacting polyisocyanates with a polyol using a catalyst and a solvent. The resulting foams have good mechanical properties, including high tensile strength and tear resistance. Polyurethane foams are also known for their good stability and resistance do deformation under load. NIPU foams, on the other hand, are synthesized without the use of isocyanate. Instead, NIPUs are synthesized using alternative crosslinking agents, such as diols, diamines, or lactones. NIPU foams generally have lower mechanical properties than traditional polyurethane foams, including lower tensile strength, compressive strength, and tear resistance.

## 3. External Blowing Agent-Based NIPU Foams

**Figure 3:**
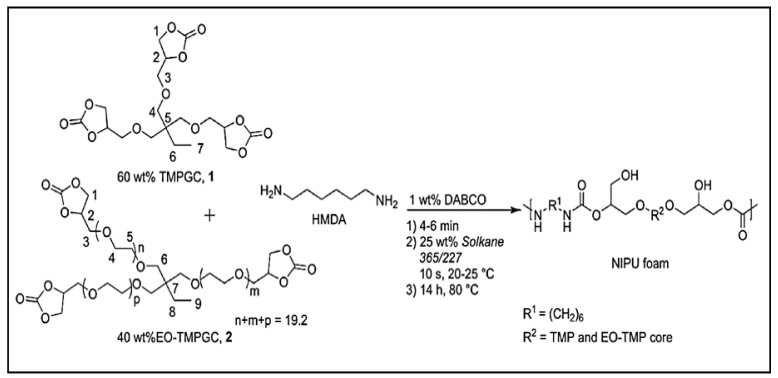
Reaction to obtain NIPUF using Solkane 365/227. Reprinted from ref [43]) with permission from the publisher.

The preparation of porous materials such as NIPUFs requires the use of blowing agents to obtain cellular NIPUs. A polymer matrix can be obtained in the reaction between cyclic carbonates or diamines and an external blowing agent for the creation of a gas—whether it is CO_2_, H_2_, or another gas—which will play a crucial role in the preparation of the desired foam. The hydrofluorocarbon liquid, commercially known as Solkane 365/227, was used as a physical blowing agent for the preparation of NIPU bio-based foams. This liquid hydrofluorocarbon is claimed to be non-flammable and has no adverse effects on the ozone layer in the atmosphere. In this study, Solkane 365/227 was added at a mass percentage of 25%. The synthesized bio-sourced cyclic carbonates, such as carbonated trimethylolpropane glycidyl ether (TMPGC) and ethoxylated TMPGC (EOTMPGC), were then mixed with the DABCO catalyst for 4–6 min at room temperature, HMDA was added, and the mixture was mixed before the addition of the blowing agent (Figure 3) [43].

Polymethylhydrogensiloxane (MH15), a blowing agent containing SiH groups that react with substances containing amines [34], has been combined with an equimolar quantity of amine to produce dihydrogen. The expansion of the materials to produce NIPU foams is caused by the release of gas. This blowing agent was used in the aminolysis of Jeffamine EDR-148 and Priamine 1073 with bi- and tricyclic carbonates obtained by the carbonation of poly(propylene oxide) diglycidether and trimethylpropan triglycidether [34]. Stefani et al. studied the effect of this blowing agent on different foam parameters and concluded that a low density of foam is produced at low blowing agent concentrations when the amine content is increased [44]. This is likely because hydrogen gas is produced when the blowing agent and amine interact.

**Figure 4:**
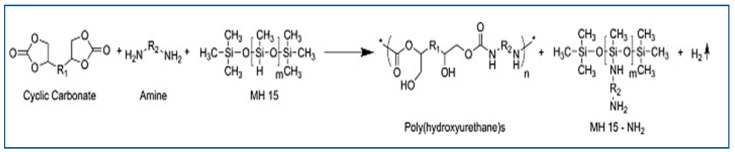
Reaction to obtain NIPUF by using MH15. Reprinted from ref [33] with permission from the publisher.

Using the same blowing agent (i.e., poly(hydromethylsiloxane) (PHMS)), a new method for the preparation of NIPU foams was developed by Valette et al., in which bio-based amine-terminated NIPU oligomers were prepared via trans-urethane polycondensation using a multi-epoxide molecule that acted as a crosslinking agent and PHMS as a surfactant and blowing agent (Figure 4) [45]. In this study, the obtained oligomers were used to create self-supported foams by reacting their amine groups with the SiH groups of PHMS, which released hydrogen gas and served as a blowing agent. Additionally, a second reaction with a multi-epoxide molecule, which served as a crosslinking agent, was performed [45].

Xuedong Xi et al. prepared glucose-based NIPU using glucose, dimethyl carbonate, a diamine, sodium bicarbonate as the blowing agent, and a silane coupling agent as compatibilizer [26] 1,8-Diazabicyclo(5.4.0)undec-7-ene. This method is convenient for obtaining rigid foams of NIPU induced by the release of CO_2_. After obtaining the polymer, sodium bicarbonate was added, and the mixture was placed in an oven at a temperature of 200 °C for 30 min to produce CO_2_, thus creating cells. It was also stated that sodium bicarbonate plays a role in rigidifying the cell walls, but no clear explanation of this effect has been presented [18]. Carbon dioxide released in this process is a safe and environmentally friendly external blowing agent that can produce NIPU foams with a uniform cell structure and good thermal insulation properties; however, the use of carbon dioxide can also result in foams with lower densities and lower compressive strength compared to foams produced with other external blowing agents.

Tannin-derived products were investigated to create NIPU foams using a blowing agent to supply foaming energy and cross-link tannin-derived products. Using various ratios of glutaraldehyde and citric acid, several tannin-based foams were created, and the mechanism of this reaction was examined using FT-IR, MALDI-TOF, and 13C NMR [20].

The properties of NIPUF can be influenced by the type of solvent and external blowing agent used in the synthesis process. Solvent can affect the properties of NIPU foams in several ways. For example, the choice of solvent can influence the solubility of the reactants and the rate of reaction. Solvents with a high solubility for the reactants can increase the reaction rate and the efficiency of the synthesis process. However, the use of certain solvents can also affect the final properties of the foam, such as its density, mechanical properties, and thermal stability. In contrast, external blowing agents are used to create the porous structure of polymer foams by generating gas bubbles during the synthesis process. Dallin et al. discussed different external blowing agent such as water, carbon dioxide, and hydrofluorocarbons used in the preparation of NIPUFs and found that the choice of external blowing agent can significantly influence the density, cell size, and thermal insulation properties of the resulting foam [46].

Each external blowing agent has advantages and limitations in the preparation of NIPU foam. Water is a cheap and readily available external blowing agent that can produce NIPU foams with a uniform and closed cell structure. However, the use of water can also lead to some limitations such as the need for high processing temperatures and the risk of hydrolysis reactions. In contrast, hydrofluorocarbons are a type of fluorinated gas that can produce NIPU foams with a more open cell structure and higher thermal insulation properties. However, hydrofluorocarbons are potent greenhouse gases and are being phased out due to their high global warming potential.

## 4. Self-Blowing NIPU Foams

Due to the weak reactivity of cyclic carbonate and amine groups at room temperature, PHU-based foams have rarely been described. Therefore, research efforts have been devoted to the development of methods for self-blowing PHU. The creation of self-blown PHU foams was proven in a recent study conducted by Anitha et al. based on selected cyclic carbonates and amines [47]. The reaction of amine-terminated oligomeric phenyl hydroxy amine (AOPHA) with cyclic carbonate of resorcinol diglycidyl ether (RDGCC) results in the release of carbon dioxide and in a parallel reaction of the hydroxyl groups and amino groups with the cyclic carbonate groups that compete during polymerization to produce PHU foams. The CO_2_ gas responsible for foaming was detected using FTIR and GC-MS [47].

**Figure 5:**
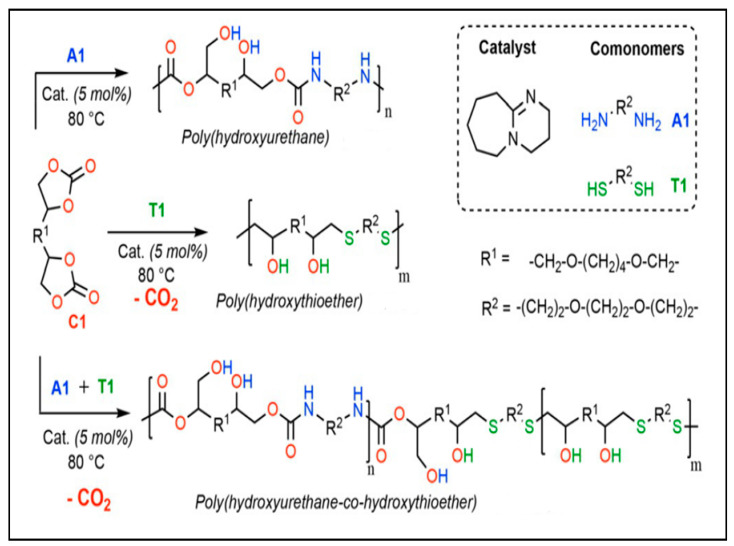
S-alkylation used to obtain NIPU foams. Reprinted from ref [48] with permission from the publisher.

The S-alkylation of thiolated monomers and tri-cyclic carbonate have been studied by Monie et al. [48] This reaction releases CO_2_ in the presence of 1,8-Diazabicyclo(5.4.0)undec-7-ene (DBU) as the catalyst (Figure 5). The high reactivity of the thiolated monomers and cyclic carbonates makes this reaction fast and efficient. Self-blowing polyhydroxythioether (PHTE) and polyhydroxyurethane foams with excellent thermal stability and mechanical properties were synthesized. Figure 5 shows the preparation of the PHTE and PHU self-blowing foams [49,50].

In a recent study, the authors described a method for producing self-blown polyurethane free-isocyanate foams by combining amines with cyclic carbonates, which create a polymer matrix, and thiolactone, which produces a thiol that reacts with a cyclic carbonate to produce the blowing agent (CO_2_) [27]. This phenomenon results in the formation of many connections such as amides, hydroxyurethanes, and thioethers inside the polymer network. This one-pot technique produced flexible and rigid foams with an open-cell structure. Figure 5 explains this method, which consists of several in situ reactions to obtain the polymer and the open cells responsible for the structure of the foam [48].

## 5. Biobased NIPU Foams

Biobased non-isocyanate polyurethane foams rely on the preparation of key intermediates such as cyclic carbonates, diamines, or polyamines, as well as CO_2_, which is used in the preparation of CC [15]. Several studies that have attempted to prepare NIPU foams with different starting materials using different methods and operating protocols are discussed in the following sections. 

### 5.1. CO_2_

**Figure 6:**
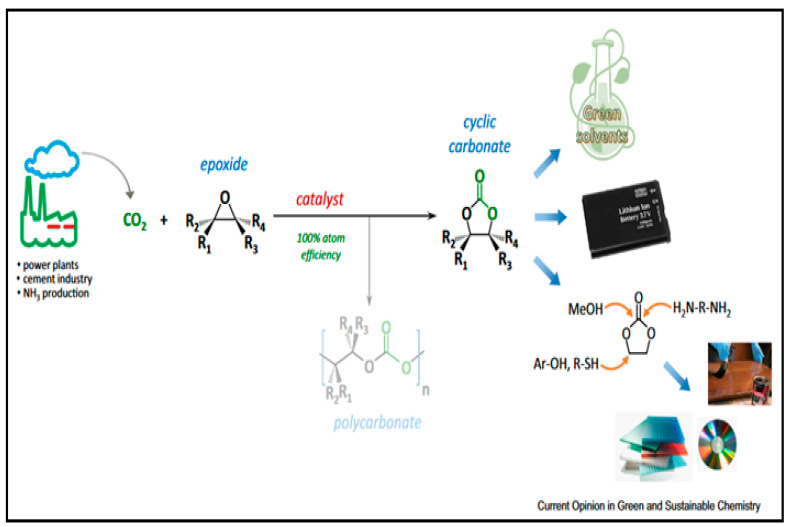
Preparation of cyclic carbonates by CO_2_ fixation and some utilizations of the cylic cabonate [54].

The use of carbon dioxide as a substitute feedstock for the synthesis of chemicals and materials has generated significant interest. The direct chemical use of CO_2_ as a co-monomer in polymerization to produce custom materials is a particularly appealing strategy (Figure 6). The catalytic copolymerization of epoxides and CO_2_ to form polycarbonates seems to be a practical technique in this case [51,52,53].

CO_2_ has been used in various studies on CO_2_ fixation with bis-epoxides. Various operating protocols and pressures have been reported for the synthesis of cyclic carbonates. McGuire et al. developed a one-step approach for combining CO_2_ with diols to form cyclic carbonates [54]. This methodology excludes the use of potent moisture-sensitive bases and allows for the use of sublimed dry ice as a CO_2_ generator. Therefore, the first CO_2_ based synthesis under atmospheric pressure of three new seven- and eight-membered cyclic carbonates under atmospheric pressure was presented [55].

The same pressure conditions were implemented in another study by R. Das and C. M. Nagaraja, which used an Ag-based catalytic system [56]. They described the sensitive synthesis of a CO_2_-philic sulfonate-functionalized UiO-66 MOF with catalytically active alkynophilic Ag(I) sites and Ag(I)-anchored sulfonate functionality, designated as MOF-SO_3_Ag. The fixation of CO_2_ involved the use of another metallic catalyst. A cobalt complex based on a carboxylic ligand with a triazole ring was investigated as an active homogeneous catalyst for the fixation of CO_2_ with epoxide. The catalyst effectively converts a variety of epoxides to industrially significant cyclic carbonates at ambient pressure without the use of solvents [57].

### 5.2. Cyclic Carbonates

Five-membered cyclic carbonates are the main components in the synthesis of NIPUF via the polyaddition reaction. The synthesis of NIPUF foam can also be carried out using other cyclic carbonates such as six-membered and seven-membered CC, but due to the toxic method used to synthesize them, investigations are more focused on five-membered cyclic carbonates. The biobased monomers used, as well as the synthesized CCs and some notes and remarks on the reaction and the types of NIPU prepared with these intermediates, are summarized in Table 1. The biobased monomers were used, as well as the synthesized CCs, and some notes and remarks on the reaction and the types of NIPU prepared with these intermediates are summarized in the Table 1.

Camara et al. synthesized three different bis cyclic carbonates using the process shown in Table 1 [57]. Butanediol bis carbonate (BBC), resorcinol bis carbonate (RBC), and trimethylpropane tris carbonate (TMPTC) were prepared through the carbonation of different epoxides. The prepared cyclic carbonates were then obtained in high yields with a carbonate equivalent weight ranging between 5.05 and 5.89 mmol/g for the three synthesized carbonates [57]. Glycidyl ethers based on trimethylpropane, glycerol, and pentaerythritol were converted to cyclic carbonates via a CO_2_ fixation reaction using tetrabutylammonium (TBAB) as the catalyst. The absorption of carbonate was detected in the 1780 cm^−1^ band, and the FTIR spectra confirmed the disappearance of the epoxy signals and appearance of new cyclic carbonate signals [58]. 

Using sorbitol glycidyl ether marketed as ERYSIS GE-60, Schmidt et al. prepared sorbitol ether carbonate (SEC) via a solventless CO_2_ fixation reaction [59]. There are two methods for the preparation of sorbitol-based cyclic carbonates using the method described above, and a second method for the preparation of sorbitol tricarbonate (STC) with DMSO as the solvent. Another method was used by Besse et al., in which isosorbide is converted to epoxidized oligo-isosorbide, and carbonation is performed to obtain a cyclic carbonate on the basis of isosorbide. Using LiBr as a catalyst, different cyclic carbonates have been prepared to synthesize PHUs with the help of different amines, such as an example 1,10 diaminodecane and JEFF400 [60].

In another approach, a tetracarbonate based on gallic acid in combination with ethylene glycol and LiBr was used to obtain satisfactory results [61]. Using another bio-based monomer, banillin, the classical epoxidation reaction using epichlorohydrin, NaOH, and TEBAC was performed to obtain vanillin bis-epoxide, followed by CO_2_ fixation using a LiBr metal catalyst and acetone as the reaction solvent [19]. Esmaieili et al. synthesized fully bio-based NIPUs using tannic acid, and the epichlorohydrin used in this study was based on glycerol and CO_2_ obtained under normal conditions [62].

### 5.3. Poly and Diamines

**Figure 7:**
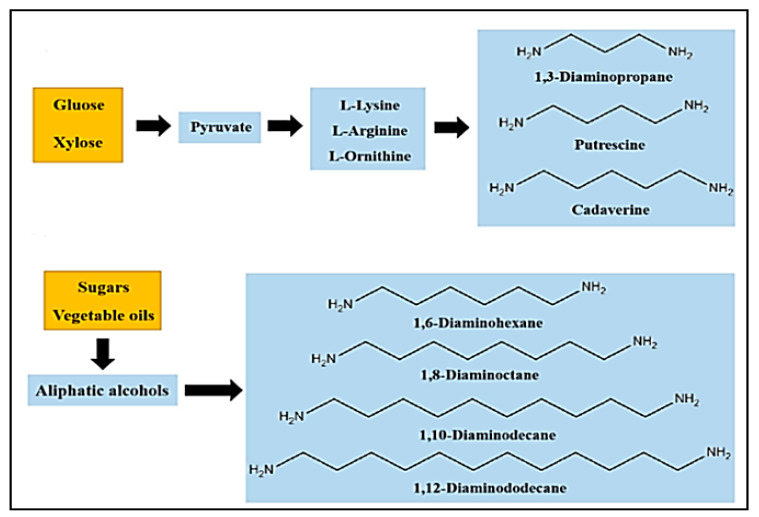
Different methods for preparing bio-based diamines. Reprinted from ref [66] with permission from the publisher.

Polyamines or diamines are used as curing agents in the polymerization and aminolysis reactions of cyclic carbonates in the presence of catalysts. These chemical compounds are responsible for the formation of the hydroxyurethane bonds. Owing to the high reactivity and nucleophilicity of amines, the choice of this diamine has a crucial role in determining the final properties of the prepared polymer. Most amines used in this synthesis are commercial and fossil-fuel-based products [63,64,65]. Wang et al. summarized the prepared diamines based on vegetable oils, sugar, and their derivatives in a recent review, and some bio-based diamines prepared using glucose, xylose, sugars, and vegetable oils are presented in Figure 7 [66].

The classification of amines by various research groups has resulted in two main amine types. The first type is modified biobased compounds such as oils, terpenes, fatty acids, and sugar derivatives; the second type is amines synthesized by biobased resources that contain one or more nitrogen atoms, such as polylysines, amino acids, chitosan, and derivatives. Using Erucic acid and oleic acid as starting materials, Nieschlog et al. successfully produced aliphatic diamines such as 1,9- nonanediamine (NDA) and 1,13-Tridecanediamine (TDA) [66]. Another study demonstrated the possibility of preparing 1,6-Hexanediamime (HMDA) using renewable resources such as succinic acid, glycerol, adipic acid, lactic acid, and levulinic acid. Vegetable oils have been used to prepare amine-functionalized molecules [67].

### 5.4. Recent Studies of Biobased Non-Isocyanate Polyurethane Foams

**Figure 8:**
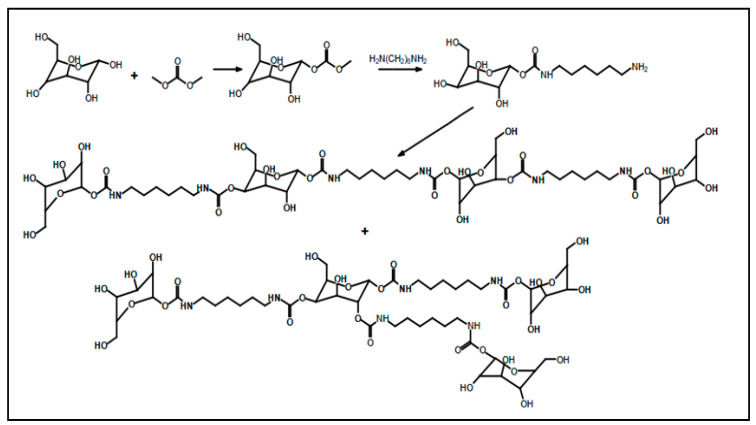
The formation of linear and branched tetramer biobased NIPUF generated from glucose. Reprinted from ref [18] with permission from the publisher.

Recent studies have been reported on the preparation of NIPU foams using biobased monomers. In a study done by Xuedong et al. in which they used glucose as the starting monomer, the aminolysis reaction is done with hexamethylenediamine and blowing agent to obtain a PHU foam [18]. Different formulations containing different ratios of Sodium Bicarbonate and Silane agents have shown different properties and flexibility of bio-based foams. Figure 8 shows the formation of tetrameric biobased NIPUFs generated from glucose [18].

The glass transition temperature and thermal stability were improved by the addition of citric acid, which acted as a crosslinker. In research using sorbitol and lysine to prepare NIPUF, it was demonstrated that the porous polymer produced had excellent thermal stability [55]. The product was utilized to produce bis cyclic carbonate under solvent-free conditions using simple diamine without solvent, as well as with no external blowing agent after being prepared using sorbitol, lysine, and dimethyl carbonate [55].

The use of a hydrolysable tannin has been studied by Azadeh et al. [68]. Tannic acid is commercially known as chestnut wood tannin. The method of preparation of NIPU foams in this study was based on three phases: the first is the preparation of the NIPU resin, then the preparation of polyurethane free-isocyanate foam, and finally, heating to cure the obtained foam. Hexamethylenediamine (HMDA) was used during polymerization with dimethyl carbonate (DMC) to obtain cyclic carbonates.

Using biomass as a feedstock for both the lignin precursors and curing agent, the protocol utilized in this study generates a highly sustainable polyurethane from lignin, resulting in products with a fully bio-based carbon content [69]. The first step consists of the oxyalkylation of kraft lignin, in which glycerol was used as a solvent and reagent, then the oxyalkylated lignin reacted with dimethyl carbonate to obtain cyclocarbonated lignin. Curing can be performed using a diamine dimer, and lignin-NIPU can then be prepared.

## 6. Outlook and Prospects

Isocyanates and polyols are frequently used in the production of PUR. However, as it is possible to minimize the use of poisonous and ecologically problematic substrates, the development of cleaner synthetic pathways, excluding diisocyanates, is a societal must. The pursuit of novel NIPUs has been accelerated by an increase in the need for safe and eco-friendly chemical precursors.

The present review has highlighted research related to non-isocyanate polyurethane foams—either by an external blowing agent or by self-blowing—and discussed the impact of different blowing agents and their effects on the density and mechanical and thermal properties of NIPUF. Even though NIPUF is entirely green and has several advantages over isocyanate-based PUF, there is still potential for advancement and development. A safer and more inventive synthetic method can be developed under moderate reaction conditions, in contrast to the majority of current NIPU, which are synthesized using cyclic carbonates at high temperatures. Because of their remarkable mechanical, thermal, morphological, and chemical properties—which are comparable to those of classical PU foams—researchers have focused on the optimization of the preparation methods of NIPUF. The aim of this study is to scale up the technology as a big step towards the successful implementation of the NIPUF on an industrial scale.

## Figures and Tables

**Table 1:** Methods of preparation of biobased cyclic carbonate.

Starting Molecules	Cyclic Carbonates Prepared	Synthesis of Epoxide Conditions	Fixation of CO_2_ Conditions	References
1,4-butanediol	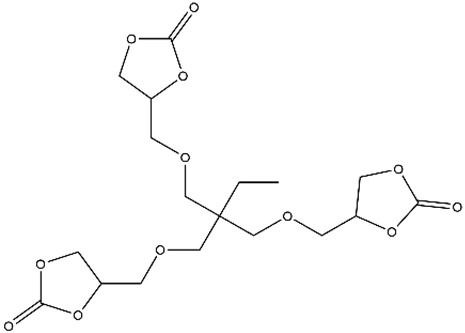	Commercial epoxy prepared by Butanediol (Butanediol diglycidyl ether)	LiBr as Catalyst DMF as solvent P = 10 bars in reactor 80 °C, 18 h	Camara et al. [57]
Glycerol	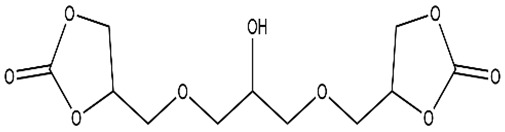	TBAB as Catalyst Solvent-free P = 30 bars in reactor 120 °C, 10 h		Fleischer et al. [58]
Sorbitol	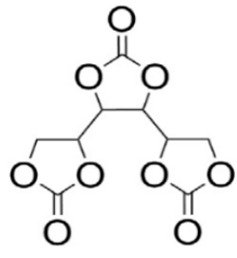	Epichlorohydrin NaOH TBAB	TBAB as Catalyst Solvent-free P = 30 bars in reactor 24 h	Schmidt et al. [59]
Isosorbide	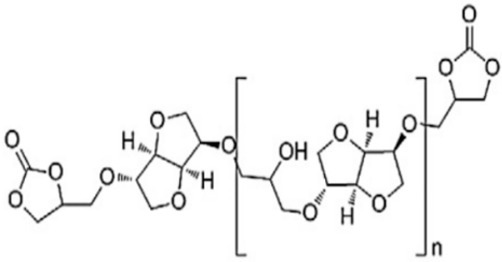	Epichlorohydrin NaOH Water	LiBr as Catalyst DMF as solvent P = 6 bars in reactor 80 °C, 12 h	Besse et al. [60]
Gallic acid	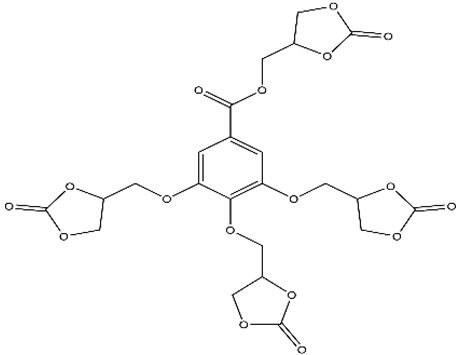	LiBr as Catalyst Ethylene glycol as solvent N-methyl pyrrolidone P = 2 MPa bars in reactor 100 °C, 9 h		Guifeng et al. [61]
Vanillin	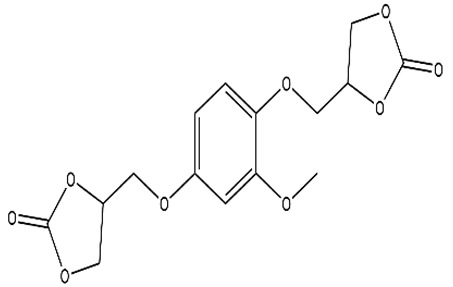	Epichlorohydrin NaOH TEBAC	LiBr as Catalyst Acetone as solvent P = 12 bars in Autoclave 80 °C, 12 h	Fache et al. [19]
Tannic acid	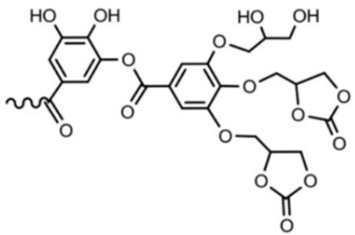	Epichlorohydrin NaOH TBAB	TBAB as Catalyst DMF as solvent CO_2_ gently bubled 80 °C, 2 h	Esmaeili et al. [62]

## Data Availability

The data that support the findings of this study are available from the corresponding author, R.V.

## Abbreviations

Polyurethane (PH), Polyurethane foam (PUF), Non-isocyanate Polyurethane (NIPU), Non-isocyanate Polyurethane foams (NIPUF), Poly(hydroxyurethane) (PHU), Poly(hydroxyurethane) foams (PHUF), Cyclic carbonate (CC), Carbone dioxide (CO_2_), Poly(methylhydrogensiloxane) (PHMS), TBAB (Tetrabutylammonium), Butanediol Bis Carbonate (BBC), Resocrinol Bis Carbonate ( RBC), Trimethylpropane tris Carbonate (TMPTC), Sorbitol Tricarbonate (STC), Sorbitol ether carbonate (SEC), Dimetyhl Carbonatre (DMC), 1,8-Diazabicyclo(5.4.0)undec-7-ene (DBU), Dimethyl sulfoxide (DMSO), Carbonated Trimethylpropane glycidyl ether (TMPGC), Ethoxylated Carbonated Trimethylpropane glycidyl ether (EOTMPGC), Polymethylhydrogensiloxane (MH15), Cyclic carbonated of Resorcinol Diglycidyl ether (RDGCC), Amine-terminated oligomeric phenyl hydroxyamine (AOPHA), Poly(Hydroxythioether) (PHTE), 1,9-nonanediamine (NDA), 1,13- Tridecanediamine (TDA), and 1,6-Hexanediamime (HMDA).
